# Biodiesel Synthesis from High Free-Fatty-Acid Chicken Fat using a Scrap-Tire Derived Solid Acid Catalyst and KOH

**DOI:** 10.3390/polym14030643

**Published:** 2022-02-08

**Authors:** Ibrahim M. Maafa

**Affiliations:** Department of Chemical Engineering, College of Engineering, Jazan University, Jazan 45142, Saudi Arabia; imoaafa@jazanu.edu.sa

**Keywords:** biodiesel, tire polymer waste, waste chicken fat, esterification, transesterification, sulfonated tire polymer char catalyst, free fatty acid conversion

## Abstract

A heterogeneous solid acid catalyst was synthesized using tire polymer waste (TPW) for the esterification of waste chicken fat (CF) enriched with fatty acids. The TPW was carbonized and functionalized with concentrated sulfuric acid under various sulfonation conditions to obtain a sulfonated tire polymer char (TPC-SO_3_H) catalyst. The TPC-SO_3_H catalyst was further characterized via acid-base titration (to ascertain the total concentration of acid), X-ray diffraction, scanning electron microscopy (SEM), energy dispersive X-ray analysis (EDAX), and Brunauer–Emmett–Teller (BET) analysis. The esterification reaction conditions of extracted chicken fat with methanol and the viability of catalyst reuse were also investigated. The composition of the free fatty acid (FFA) decreased to below 1% under optimum reaction conditions of 5% TPC-SO_3_H catalyst, the methanol-to-CF molar-ratio of 15:1, and a reaction time of 120 min at 70 °C. The catalyst preserved its conversion efficiency above 90%, even after three cycles. The results demonstrate that the catalyst is applicable and efficient in the esterification of raw materials containing various fatty acid compositions since different carbonized materials have distinct abilities to combine acid groups. Furthermore, after de-acidification of CF-FFA by the as-prepared TPC-SO_3_H catalyst, the neutral CF was transesterified completely to biodiesel and characterized via Fourier Transform Infrared (FTIR) spectroscopy, proton nuclear magnetic resonance (^1^H NMR) spectroscopy and physicochemical analysis. This work unveils a promising technique for utilizing tire waste generated in large quantities for the development of a novel heterogeneous acid catalyst for biodiesel production.

## 1. Introduction

Biodiesel is an eco-friendly and efficient fuel, that is regarded nowadays as an alternate “direct-pour” fuel to petroleum-derived diesel. It works efficiently in almost all available contemporary diesel engines without requiring any significant modification to them. It is an emerging and promising fuel candidate that may open up new avenues to reduce our sole dependence on fossil fuels. It has better lubrication properties than petroleum diesel which can significantly suppress engine wear. Moreover, it has higher oxygen content than petroleum diesel which results in lower environment-polluting emissions, thus curbing global warming [1]. The higher oxygen content of biodiesel ensures its complete combustion in the diesel engine, which leads to a remarkable decrease in toxic air polluting emissions such as carbon monoxide and particulate matter throughout the engine load range [2]. However, cold weather poses a cold flow problem in biodiesel which thwarts its performance [3,4]. For biodiesel to exhibit better performance and stability at accepted standards at low-temperatures, it should comprise comparatively low concentrations of saturated fatty acid methyl esters (FAME) and poly-unsaturated FAME [5]. There are various techniques to improve the poor cold-flow characteristics of biodiesel: blending with additives (pour point depressant) [6,7], winterization [8], blending with other diesel fuels in the right proportion [9], mixing with branched-chain esters [10], and blending with vegetable oils with low crystallization temperatures [11], etc. Sanjeevannavar et al. employed hydrogen peroxide (H_2_O_2_) as an additive in the biodiesel synthesized from Jatropha oil via transesterification in the presence of a catalyst to evaluate the performance of a four-stroke single-cylinder diesel engine [12]. They reported that blending the biodiesel with H_2_O_2_ furnished additional hydrogen and oxygen that ensured complete combustion of the fuel and curbed the emissions. The blend comprising 60% diesel, 30% biodiesel, and 10% H_2_O_2_ exhibited a 20% enhancement in brake-thermal efficiency (BTE) while exhibiting a 25% decrement in brake-specific fuel consumption (BSFC) as compared to the blend comprising 60% diesel and 40% biodiesel. They also observed that the emissions of hydrocarbon (HC), smoke, and CO_2_ reduced by 17.54%, 24.6%, and 3.53%, respectively, while the NO_x_ emission increased. Mujtaba et al. investigated the influence of various alcohols as oxygenated additives on engine performance and emissions [13]. They considered various test fuel blends and tested on a single-cylinder four-stroke, direct-injection diesel engine at various speeds and 100% load. Their study suggested that the blended fuel comprising 70% diesel, 20% palm biodiesel, and 10% bioethanol exhibited the best performance among all the employed bioethanol-based blends by enhancing the BTE and BSFC significantly while reducing the NO_x_ emission. Moreover, this fuel blend ameliorated the neat diesel cloud point. More recently, researchers also attempted to ameliorate the biodiesel properties and enhance its performance by adding nanoparticles in the form of additives [14,15,16,17,18]. They demonstrated that adding oxygenated, metallic, non-metallic, and organic nanoparticles with petroleum diesel–biodiesel fuel blends improved the thermophysical characteristics of the fuel, fuel mixture stabilization, and the associated heat transfer rate. It also improved the engine performance and reduced harmful exhaust emissions.

Biodiesel combustion enhances the emission of nitrogen oxides, which pose potential threats to human health and the environment [19]. Various researchers have studied the toxicological effects of biodiesel and its blends on human health [20,21,22,23,24,25,26]. Liu et el. assessed the toxicity of emissions from a diesel engine running on biodiesel [27]. They tested the engine employing diesel and biodiesel blended fuels in the ratios of 10, 30, 50, 75, and 100% of biodiesel by volume, while operating the engine at idle state and 10, 33, and 55% loads. Their analysis revealed that emissions of carcinogenic carbonyl compounds increased when biodiesel fuels were employed. About 70 to 90% of all carbonyl emissions were made-up of formaldehyde, acetaldehyde, acrolein, and acetone. They observed that the concentrations of CO_2_ and NO_x_ from the biodiesel blend comprising 10% biodiesel were nearly the same as that of diesel. Also, it posed a higher degree of toxicity and cytotoxicity than diesel, suggesting that blending with biodiesel might cause adverse health issues due to toxic gaseous emissions.

Feedstocks such as animal fats, vegetable oils, waste cooking oils, and grease are generally employed for biodiesel production [1]. Biodiesel may be defined as animal fat- or vegetable oil-based diesel fuel comprising of a long chain of alkyl (such as methyl, ethyl, or propyl) esters [28]. Biodiesel contains FAME which are built of unsaturated “olefin” components; in this case, biodiesel is synthesized by employing ethanol instead of methanol, the resultant molecules are fatty acid ethyl esters (FAEE). Biodiesel can be synthesized either via transesterification of vegetable oils or by direct esterification of animal fats with alcohols such as methanol, ethanol, and propanol. Generally, biodiesel production requires a catalyst either a homogeneous catalyst or heterogeneous catalyst to speed up the chemical reaction. Various homogeneous acid catalysts have been employed for the esterification process, such as hydrochloric acid (HCl), nitric acid (HNO_3_), sulfuric acid (H_2_SO_4_), and phosphoric acid (H_3_PO_4_), but they pose several drawbacks including that they are irrecoverable, unstable, make the purification step complex and corrode the equipment. To address these issues, various researchers have proposed the utilization of heterogeneous solid acid catalysts [29]. These catalysts are comparatively more effective in biodiesel production since they incur low processing costs, can be recovered for reuse, and pose minimal damage to the environment without calling for any sophisticated purification procedures [30]. To attain the low production cost of biodiesel, researchers have employed various inexpensive feedstocks such as karanja oil, waste cooking oil, animal fat (AF), palm fatty acid distillate (PFAD), sludge oil, grease, etc. However, these types of feedstocks contain a high amount of FFAs which deems them not suitable to be used with basic catalysts since basic catalysts will create saponification issues [31]. Thus, utilization of heterogeneous solid acidic catalysts such as sulfonated carbohydrate-based catalysts [32,33,34,35,36], sulfonated carbon-based catalysts [34,37], sucrose-derived carbon catalysts [38], and jatropha curcas biomass catalysts [31] have been extensively researched, primarily because of their high porosity, low-cost, and abundant availability. More recently, the utilization of solid acid sulfated metal oxide catalysts [29], namely, sulfated zirconia oxide [39], sulfated tin oxide [40], and sulfated iron oxide [41] have also been investigated. These sulfated metal oxide catalysts exhibit strong acidic features because of the existence of the sulfate functional group [42].

Lokman et al. performed esterification of PFAD by using a sulfonated-glucose solid acidic catalyst and obtained 94.5% FAME conversion at a reaction temperature and time of 65 °C and 134 min, respectively [43]. Chin et al. also reported the esterification of PFAD by utilizing sulfonated sugarcane bagasse catalyst and realized 80% FAME at a reaction time and temperature of 30 min and 170 °C, respectively [34]. More recently, Thushari et al. investigated the effectiveness of sulfonated coconut meal residue (CMR)-derived solid acidic catalyst synthesized via esterification of waste palm oil (WPO), and obtained 92.7% of FAME at a reaction temperature and time of 65–70 °C and 720 min, respectively [44].

In this study, we report a novel strategy to produce a high-capacity and cost-effective catalyst utilizing waste tires for biodiesel synthesis from waste chicken fat. The main composition of tires is solid rubber, which is very durable and non-biodegradable, which presents challenges for their disposal. It is a long-established tradition to dispose of waste tires in landfill and by illegally dumping, which, poses a severe hazard to the environment and human health [45,46,47]. According to statistical data, approximately 926 million scrap tires are discarded in China annually, while in USA and Saudi Arabia the data records annual disposal of 242 million tires [48] and 20 million tires, respectively. These quantities of scrap tires will continue to increase rapidly with the inevitable growth of motor vehicle industries in the future. Thus, there is a dire necessity to turn our research focus to the scientific disposal of waste tires, to alleviate environmental pressure and reduce pollution, whilst recycling tire scrap with maximum efficiency [49]. Currently, the primary recycling methods for waste tires include re-treading, ambient mechanical grinding, cryogenic grinding, direct incineration, and pyrolysis [47,50,51,52]. Among these technologies, pyrolysis is considered the most economical and efficient way to treat waste tires because it can completely decompose them into beneficial by-products, like value-added pyrolysis gas and oil [51]. Nevertheless, the waste tire pyrolysis residue (WTPR) that remains after separation limits the economic benefits. WTPR, which accounts for approximately 40% of the total amount of pyrolysis products, mainly includes carbon, inorganic components (ash), and macromolecular rubber hydrocarbons. Apart from the said by-products, there is also the generation of gaseous pollutants carrying nitrogen, sulfur, and chlorine [53]. The chlorinated pollutants like HCl and dioxins are highly corrosive and toxic [54]. Moreover, extracting the ashes and sulfides from the by-products involves extra cost and effort, which limits the cost-effectiveness of the pyrolysis process.

To overcome all the above-mentioned drawbacks involved with the conventional recycling methods for tire waste, herein we propose a novel approach to utilize the tire polymer waste (TPW) for synthesizing high-capacity and cost-effective sulfonated tire polymer char (TPC-SO_3_H) catalyst which can efficiently synthesize biodiesel out of chicken fat. We demonstrate that this method of synthesizing biodiesel is the best approach from a green chemistry perspective. Moreover, this method delivers two-fold benefits; first, it solves the ever-pressing problem of safe tire scrap disposal and second, it produces highly sought after biodiesel fuel that is competent for the future transportation industry.

## 2. Experimental Setup and Methodology

### 2.1. Preparation of a Sulfonated Tire Polymer Char (TPC-SO_3_H) Catalyst

**Figure 1:**
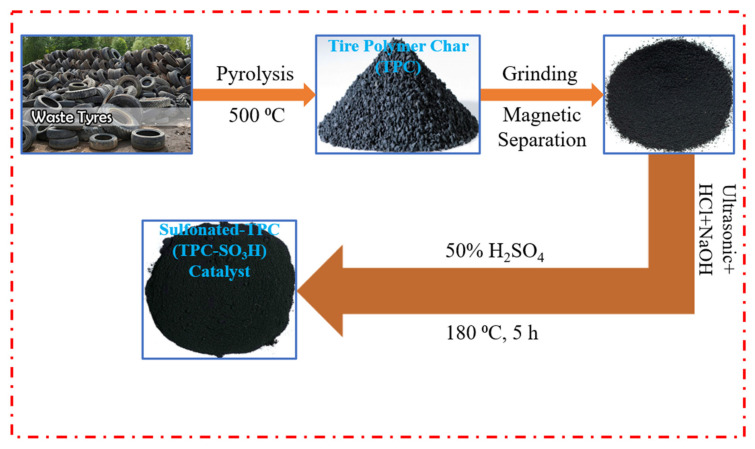
Sulfonated tire polymer char (TPC-SO_3_H) catalyst synthesis from tire polymer waste (TPW).

All the materials and reagents utilized in the experiments were of analytical grade. The chemicals KOH and HCl were procured from Loba Chemie Pvt. Ltd. (Mumbai, India), while H_2_SO_4_ was procured from Merck Chemicals (Darmstadt, Germany). The chemicals were used as received without any purification. The catalyst preparation followed the following steps: (i) tire polymer waste (TPW) was mechanically ground in a crusher machine (Wanrooetech, Zhangjiagang, China) at an ambient temperature, followed by washing and then drying at 90 °C overnight, (ii) the crushed TPW was then pyrolyzed at 500 °C for 4 h in a nitrogen gas atmosphere, (iii) the produced tire polymer char (TPC) was exposed to a magnetic field to separate the iron present in it and then further ground to fine powder, (iv) ultrasonic-assisted chemical treatment of pure TPC with acid (HCl) and alkali (NaOH) was then performed to remove metals and silica, respectively, (v) the residue was further washed with deionized water to achieve neutrality and then left to dry at 80 °C overnight, (vi) the dried TPC was finally sulfonated via a one-step sulfonation process employing concentrated H_2_SO_4_ (50%) [12]. The sulfonation was performed by gradually adding 100 mL concentrated H_2_SO_4_ drop-by-drop to a sample of 10.0 g dried TPC in a 500 mL beaker and then by subjecting the solution to continuous stirring for a duration of 5 h at 180 °C. The solution was allowed to cool to room temperature, filtrated, and washed with hot deionized water to remove the excess H_2_SO_4_ until the solution became neutral. The precipitated black solid was oven-dried at 90 °C for 24 h to produce the TPC-SO_3_H catalyst. The complete procedure of synthesizing the catalyst is illustrated in Figure 1.

### 2.2. Biodiesel Synthesis

#### 2.2.1. Chicken Fat Extraction

The waste chicken fat (CF) was purchased from a local chicken market and washed with tap water to remove undesired bones, blood tissues, and dust particles. A sample of 200 g of chicken fat was loaded into the microwave oven and heated at 90 °C for 300 min, and approximately 130 g of chicken fat was extracted. After the lubrication process, the melted CF and chicken waste were separated using a textile filter. The extracted melted fat was mixed with 50 g of anhydrous Na_2_SO_4_ and the solution was left for 50 min to dry completely, thereby removing residual water. The CF was then washed with hot water two to three times to remove the collagen substances and then heated above 100 °C to complete drying. An acid value test was conducted on the extracted chicken fat. The acid value of an oil is defined as,

(1) $$\mathrm{Acid}\;\mathrm{Value}\;\left({\mathrm{mg}\;\mathrm{KOH}\;g}^{-1}\right)=\frac{56.1\times \;V\;\times \;C\;}{m}$$

where, C is the KOH solution concentration (mol/L), V is the volume of KOH solution (mL), m is the chicken fat weight (g).

The composition of the free fatty acids in the CF is defined as,

(2) $$\;\mathrm{FFA}\;\left(\%\right)=\frac{\mathrm{Acid}\;\mathrm{Value}}{2}$$

According to Equation (1), the obtained acid value of extracted chicken CF was 27.4 mg KOH g^−1^ which according to Equation (2) corresponds to 13.7% of FFA. Since the FFA value was greater than 1, the extracted oil was not directly subjected to a transesterification reaction, rather its esterification was first conducted to reduce the acid value.

#### 2.2.2. Esterification and Transesterification

**Figure 2:**
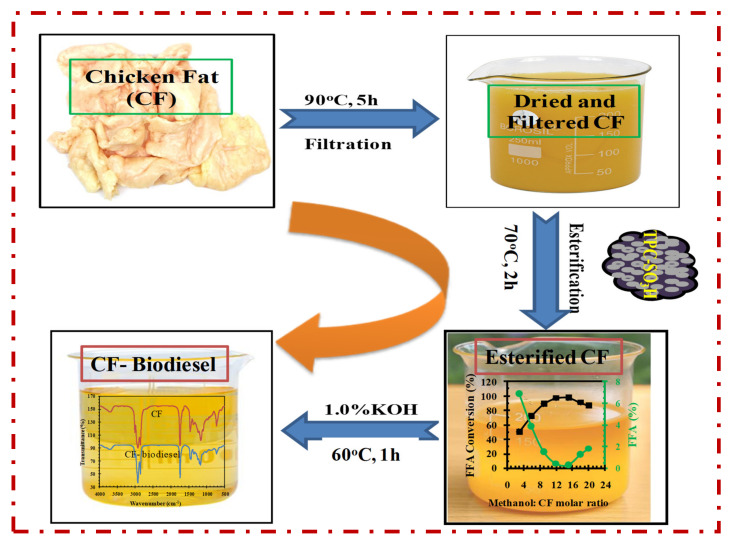
Biodiesel synthesis via two-step conversion process.

Owing to the high FFA level (13.7%) in the extracted chicken fat, we subjected it to a pre-treatment esterification step in the presence of our as-synthesized sulfonated tire polymer char (TPC-SO_3_H) catalyst to reduce FFA% to 1 and to avoid saponification. To perform esterification, a certain amount of extracted chicken fat was mixed with a specified quantity of methanol (CH_3_OH) and as-synthesized catalyst. The temperature of the mixture was maintained at 70 °C by employing an oil bath, and the mixture was continuously stirred at 300 rpm during the entire 120 min experimental run. After esterification, the catalyst was isolated from the esterified fat by employing a centrifuge spinning at 3000 rpm for 20 min. Further, to remove residual water from the produced esterified oil it was mixed with Na_2_SO_4_ in a conical flask for 50 min at 90 °C. A range of experiments was conducted by varying reaction conditions including reaction time ranging from 15–160 min, reaction temperature ranging from 40–90 °C, methanol-to-CF molar ratio ranging from 3–20, and catalyst dosage ranging from 1–10 wt.% of the chicken fat. During the second step of the final biodiesel conversion, the esterified CF was exposed to the transesterification reaction wherein a mixture of esterified fat and methanol in a molar ratio of 6:1 (methanol: CF) was treated with an alkaline catalyst KOH (1.0 wt.%) at 60 °C for 60 min duration. The complete process of biodiesel synthesis has been shown in Figure 2. After the biodiesel synthesis, production efficiency at each stage was determined by using the following Equation (3),

(3) $$\mathrm{Biodiesel}\;\mathrm{Yield}\;\left(\%\right)=\frac{\mathrm{Weight}\;\mathrm{of}\;\mathrm{biodiesel}\;}{\mathrm{Weight}\;\mathrm{of}\;\mathrm{chicken}\;\mathrm{fat}\;}\times 100$$

### 2.3. Characterization Techniques

The XRD patterns of the synthesized TPC-SO_3_H catalyst were obtained employing a Shimadzu XRD 6000 X-ray Diffractometer (Kyoto, Japan) equipped with a Copper K-α X-ray radiation source corresponding to an X-ray wavelength of 1.542 Å, and running at an operating voltage and current of 40 kV and 30 mA, respectively.

Surface morphology characterization of the TPC-SO_3_H catalyst was performed by scanning electron microscopy (SEM) technique employing a Jeol JSM-7000F FE-SEM (Osaka, Japan). A small quantity of powder was strewn onto double-stick carbon tape mounted on an aluminum sample holder.

The specific surface area of the TPC-SO_3_H catalyst was determined by employing the N_2_ gas adsorption technique developed by Brunauer, Emmett, and Teller (BET) using a porosimeter, model 3200E YOUNG, Quantachrome (UK). The pore volumes and pore diameters were determined by the theory developed by Brunauer, Joyner, and Halenda (BJH).

Fourier transform infrared (FTIR) spectra of the chicken fat (CF) and CF-derived biodiesel were recorded on a PerkinElmer Spectrum 100 FT-IR Spectrometer (USA) fitted with an exclusive universal attenuated total reflection (UATR) polarization accessory. The FTIR spectra were recorded in the wavenumber range from 4000–500 cm^−1^ at a resolution of 4 cm^−1^. We reported the spectra with the average over 16 scans.

A Bruker Avance-III 300 MHz NMR Spectrometer (Bruker, Milan, Italy) was used to capture the ^1^H-NMR spectra of the chicken fat (CF) and CF-derived biodiesel. Deuterated chloroform (CDCl_3_) and tetramethylsilane were employed as a solvent and the internal standard, respectively. The transesterification reaction was monitored via ^1^H-NMR spectroscopy. The following equation describes the conversion:

(4) $$C=100\times \frac{2A_{\mathrm{Me}}}{3A_{\mathrm{CH}2}}$$

where, C = percentage conversion of triglycerides to corresponding methyl esters., A_Me_ = integration value of the methoxy protons of the methyl esters (3.66 ppm), A_CH2_ = integration value of methylene protons (2.26 ppm).

## 3. Results and Discussion

### 3.1. Catalyst Characterization

Waste tires pose a great danger to the environment if burned in the open air because they contain a high amount of sulfur, and on burning they emit a high amount of harmful oxides, such as sulfur and nitrogen oxides, and carbon monoxide. Thus, the treatment of waste tires by pyrolysis is considered an ideal and safe way to treat this waste. The pyrolysis of tires produces three products: oil, char, and gas. The char obtained from the pyrolysis contains a large amount of inactivated carbon and oxygen and needs chemical treatment to activate the surface of the carbon to acquire acidic groups that can convert the free fatty acids in chicken fat into biodiesel.

**Figure 3:**
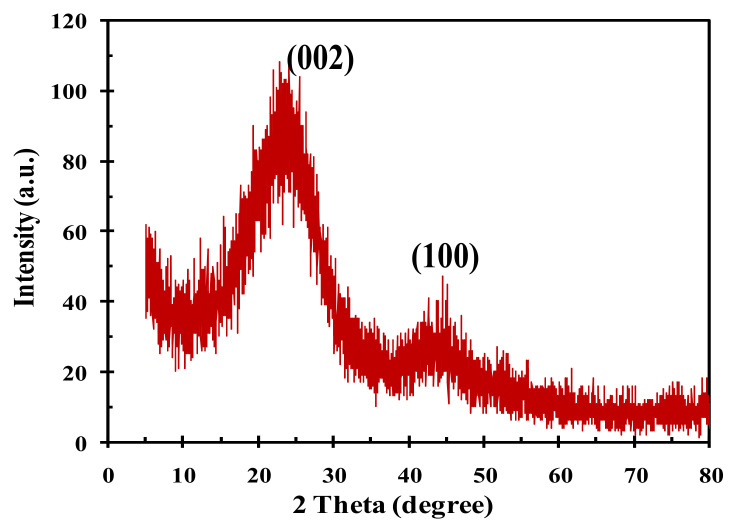
XRD pattern of the sulfonated tire polymer char (TPC-SO_3_H) catalyst.

The X-ray diffraction pattern of the catalyst sample is displayed in Figure 3. The sample exhibited high background intensity, which indicates that the carbon phase comprised of highly disordered matter in the form of amorphous carbon. The diffractogram shows the presence of an asymmetric band peak (plane 002) around 23.5°, which indicates the separation of the aromatic ring layer, in addition, the carbon also includes other structures such as graphite. The second peak (plane 100) can be attributed to disordered carbon. These observations provide a plausible suggestion that the carbon has an intermediate structure between the graphite and amorphous state called a “turbostratic” structure [55].

**Figure 4:**
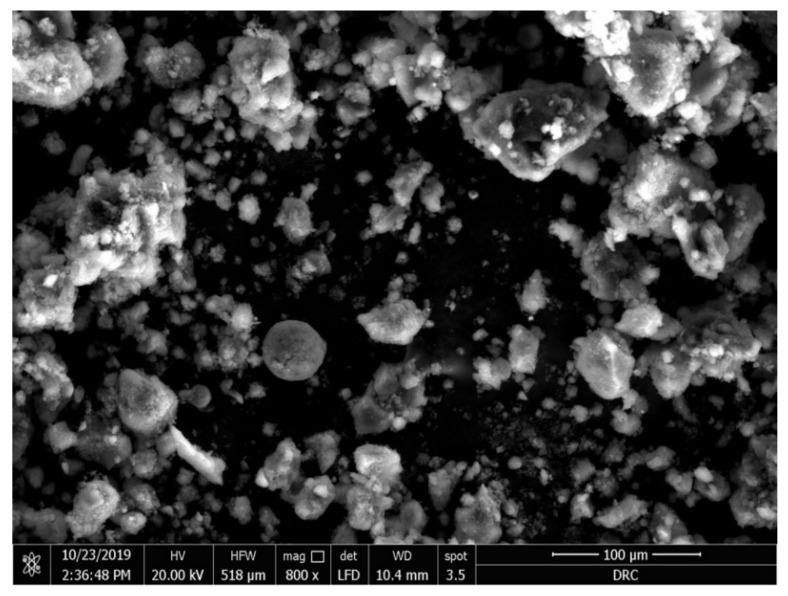
SEM image of the sulfonated tire polymer char (TPC-SO_3_H) catalyst.

The SEM image of the as-prepared TPC-SO_3_H catalyst is shown in Figure 4. The acid catalyst consists of irregular particles of various diameters ranging from 3 to 80 µm. We conclude that the large, aggregated particles could have been formed due to the agglomeration by solid-state reactions during the sulfonation process.

**Figure 5:**
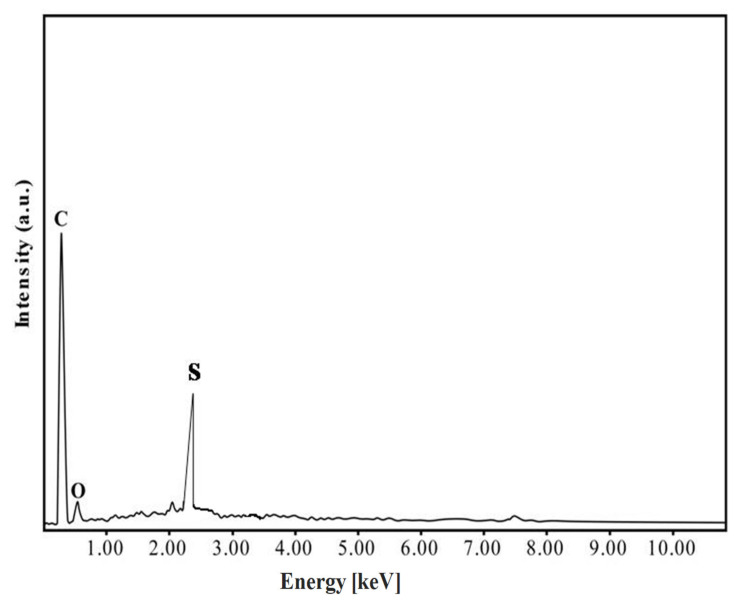
EDAX image of the TPC-SO_3_H catalyst.

The chemical composition of the TPC-SO_3_H catalyst was confirmed by the energy dispersive X-ray spectrometry technique (EDAX), as displayed in Figure 5. From the EDAX result, we observe the presence of C, O, and S elements in the TPC-SO_3_H catalyst.

**Figure 6:**
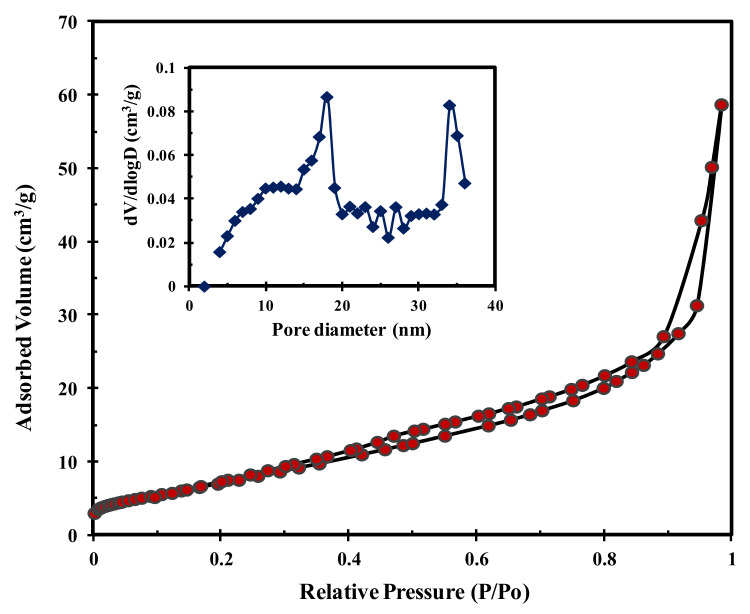
BET and pore size distribution of TPC-SO_3_H catalyst.

The TPC-SO_3_H catalyst pore size distributions and N_2_ adsorption-desorption isotherms obtained at −196 °C are shown in Figure 6. The isotherm generated by the TPC-SO_3_H exhibited a narrow hysteresis loop with increasing pressure over the P/P_0_ range of 0.6–1.0, which confirms a typical type-IV isotherm and approximately 79.8 m^2^/g BET surface area. The TPC-SO_3_H pore size distribution is summarized in the inset of Figure 6. There are two distinct mesoporous structures with pore sizes ranging between 17.6 nm and 34.2 nm on average. The corrosive effect of concentrated sulfuric acid on the pore walls of the tire might explain the presence of the bimodal nanostructure of the catalyst.

### 3.2. Biodiesel Production

The production of biodiesel is divided into two stages. The first step is to reduce the FFA by converting it into biodiesel in order to avoid saponification during the alkali transesterification stage. The second step involves the conversion of CF-triglyceride to FAME utilizing an alkali catalyst (KOH) via the transesterification process.

#### 3.2.1. Acid Catalyzed Chicken Fat Esterification (1st Stage)

The effect of reaction time, temperature, methanol-to-CF molar ratio, and catalyst loading on acid catalyzed chicken fat esterification were investigated.

**Figure 7:**
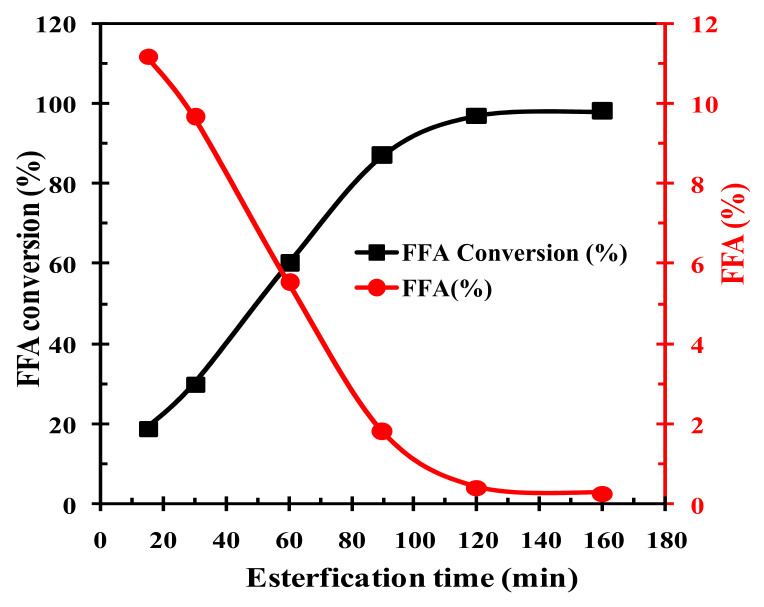
Effect of the reaction time on FFA conversion to FAME and deacidification.

The effect of the reaction time on chicken fat esterification is illustrated in Figure 7. The reaction was conducted with a methanol-to-CF molar ratio of 15:1, catalyst dosage of 5 wt.%, at 70 °C, and reaction time varying from 15–160 min. The FFA percentage decreased from 11.2% to 0.276%, while the FFA to FAME conversion increased from 19% to 98.8% with increasing time until 120 min. It is noteworthy that the system reached equilibrium at 120 min, beyond which both the quantities remained almost constant. Conclusively, the reaction time of 120 min produced the highest FFA deacidification (0.276%).

**Figure 8:**
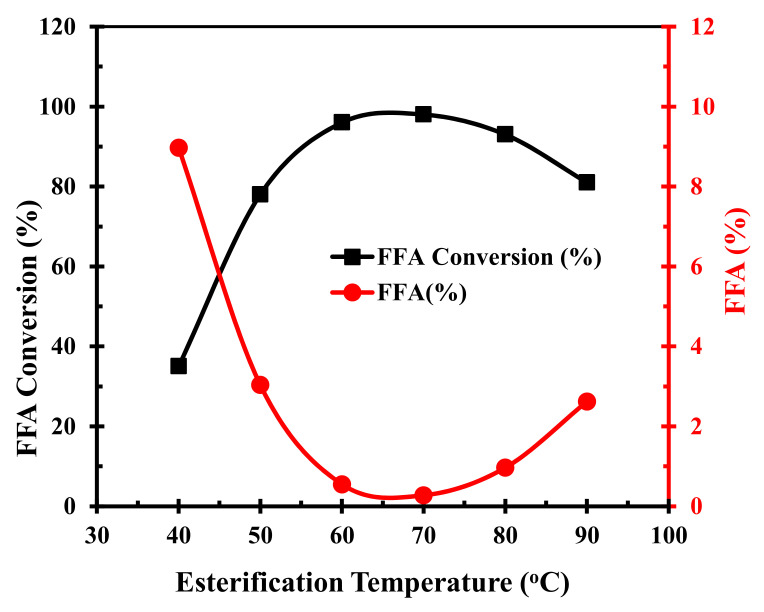
Effect of the reaction temperature on FFA conversion to FAME and deacidification.

The reaction temperature played an important role in the conversion process of chicken fat to FAME. The effect of temperature on FFA conversion was investigated by conducting experiments at temperatures varying from 40–90 °C in steps of 10 °C, while the other parameters, namely, methanol-to-CF molar ratio, catalyst dosage, and reaction time were fixed at 15:1, 5 wt.%, and 120 min, respectively, as shown in Figure 8. We observed that increasing the reaction temperature from 40–70 °C led to an increase in conversion from 35 to 98.8%, and beyond this temperature, the conversion decreases. We surmise that the collision rate of the reactant molecules was enhanced via high energy input due to temperature increase, which accelerated the chemical reaction and led to high FFA conversion. However, the FFA conversion cannot be attributed solely to the temperature increase, rather it is also related to factors such as limited mass transfer between the catalyst and reagents, and the adverse effects of methanol vaporization at higher temperatures. Thus, the conversion efficiency decreases beyond 70 °C. Therefore, we found that 70 °C was the most effective temperature for FFA conversion.

**Figure 9:**
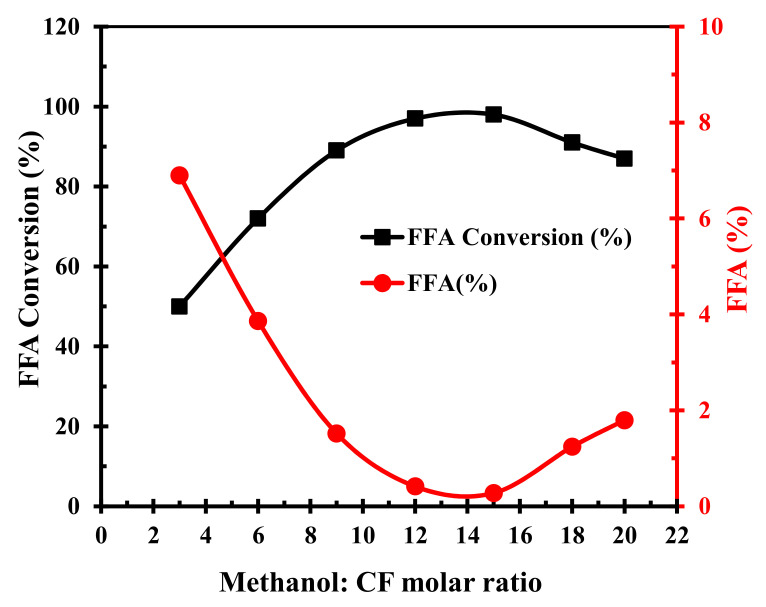
Effect of methanol-to-CF molar ratios FFA conversion to FAME and deacidification.

The methanol-to-CF molar ratio is among the critical factors that affect the conversion efficiency and the production cost of biodiesel. Theoretically, the esterification reaction requires one mole of methanol for each mole of FFA. However, practically, the methanol should be more than the FFA to drive the reaction towards completion as the esterification of FFA with methanol is reversible. To probe the effect of methanol-to-CF molar ratios on esterification, various experiments were conducted varying this ratio from 3–20 while maintaining 70 °C temperature for 120 min duration employing a catalyst dosage of 5 wt.%. The FFA conversion increased from 50% to 98.8% when the methanol-to-CF molar ratio increased from 3:1 to 15:1, beyond which it rapidly decreased, as shown in Figure 9. Thus, the highest esterified yield (98.8%) was obtained at 15:1 methanol-to-CF molar ratio.

**Figure 10:**
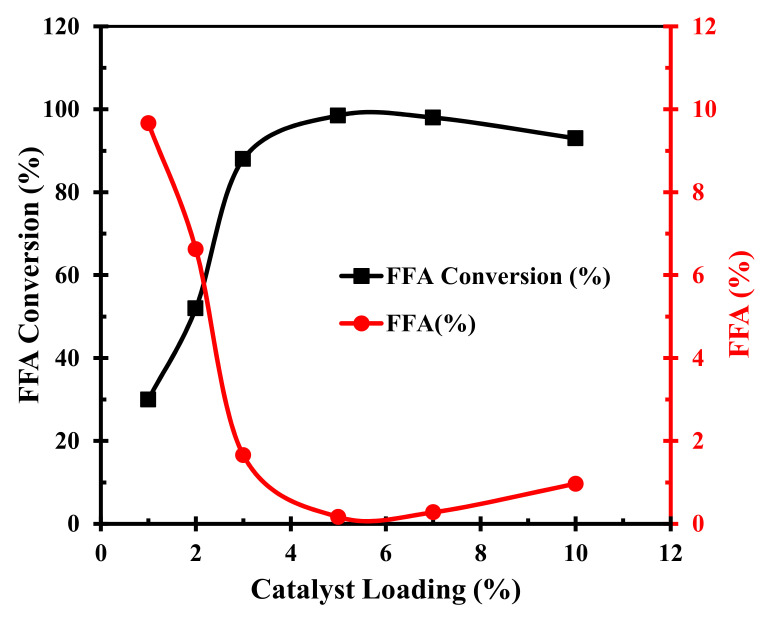
Effect of catalyst concentration on FFA conversion to FAME and deacidification.

Lastly, we investigated the influence of the TPC-SO_3_H solid catalyst dosage fed into the reaction on the conversion efficiency of FFA to FAME. The catalyst loading in the esterification was varied from 1 to 10 wt.% while maintaining 70 °C temperature for 120 min duration, with a 15:1 methanol-to-CF molar ratio. The FFA conversion increased rapidly from 30% to 98.8 % when the catalyst dosage increased from 1 to 5 wt.%, however, further increasing the catalyst dosage decreased the conversion efficiency, as displayed in Figure 10. We present a conjecture that adding an excessively high amount of the TPC-SO_3_H solid catalyst into the reaction system decreases the solubility of the methanol and CF, hence limiting the mass transfer. Therefore, we conclude that the maximum biodiesel was produced at 5 wt.% catalyst dosage.

#### 3.2.2. Reusability of TPC-SO_3_H Catalyst

**Figure 11:**
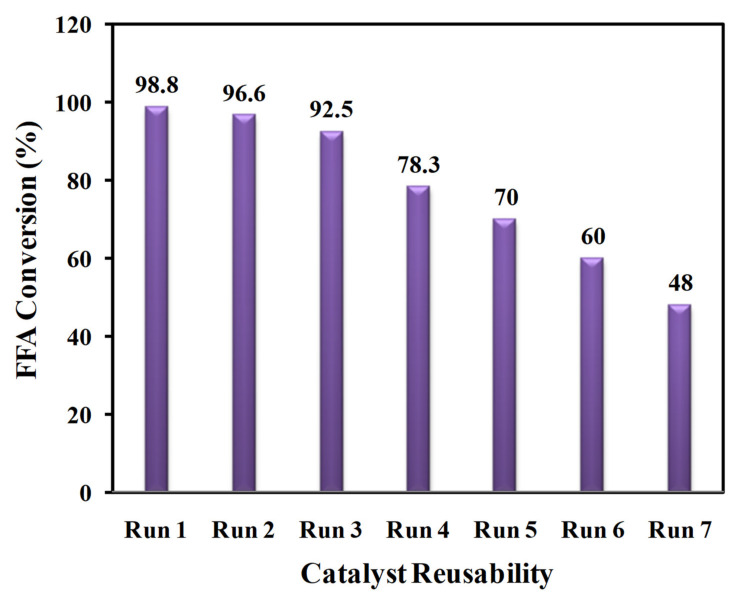
Reusability tests of the TPC-SO_3_H catalyst for seven experimental runs under optimal conditions.

The prominent merit of employing solid acid catalysts is that they can easily be recovered from the reaction mixture and reused over various reaction cycles. We studied the tenor of the activity of regenerated solid acid TPC-SO_3_H catalyst by conducting esterification runs seven times sequentially under optimum reaction conditions at 70 °C and 120 min with a 15:1 methanol: CF molar ratio, and a catalyst dosage of 5 wt.%, as ascertained previously. It can be inferred from Figure 11 that there is a continuous loss in the activity of the catalyst with acceptable reusability up to three cycles. The steady and gradual death of the active sites in each run may explain the gradual decline in the catalytic activity during the latter two runs of the first three cycles. However, we notice that after the third cycle of repeated use, the catalytic activity decays dramatically. A few plausible explanations for this significant activity waning are as follows: (i) the weak bonding of -SO_3_H groups with the carbon structure may lead to leaching of these active sites, (ii) the -SO_3_H, –OH, and –COOH groups present stable Brønsted sites because of which these sites have a high propensity to absorb water from the reaction mixture, which in turn prevent the reactants from engaging with the acidic sites, (iii) the accumulation of the reactants might deactivate the catalyst active sites, and (iv) the morphology of the catalyst alters due to continuous reuse up to the third cycle which may have disintegrated the carbon structure; in such case, the pore size of the carbon structure becomes smaller preventing the reactants from interacting with the sulfonic and acidic sites.

#### 3.2.3. Catalytic Activity Comparison of the Waste-Derived Sulfonated Char Catalysts

We have already established that the optimized reaction conditions for the TPC-SO_3_H catalyzed esterification of CF is 70 °C reaction temperature, 120 min reaction time with a methanol-to-CF molar ratio of 15:1, and a catalyst loading of 5 wt.%. Under these optimal settings, the conversion of FFA to FAME was 98.8%. The sulfonated tire polymer char (TPC-SO_3_H) catalyst synthesized in the present work exhibits excellent catalytic performance toward CF enriched with fatty acids. The efficiency of this catalyst surpassed other formerly reported bio-waste-derived sulfonated carbon-based solid acid catalysts, as summarized in Table 1.

#### 3.2.4. Alkali Catalyzed Transesterification (2nd Stage)

The esterified CF utilized in alkali-catalyzed transesterification contains 0.29 wt.% FFA. The decrease in the FFA content makes it ideal for transesterification using an alkali catalyst. The transesterification was performed utilizing 1.0 wt.% KOH as a catalyst with 6:1 methanol-to-CF molar ratio at a reaction temperature of 60 °C for a duration of 1.5 h [62], yielding the FAME (biodiesel) conversion of 98.7%.

### 3.3. Biodiesel Characterization

The physicochemical properties of the biodiesel derived from chicken fat (CF) were analyzed and compared to the European Standard EN 14214, as summarized in Table 2. The table confirms that the physicochemical characteristics of the produced biodiesel (FAME) satisfy the EN 14214 standards.

**Figure 12:**
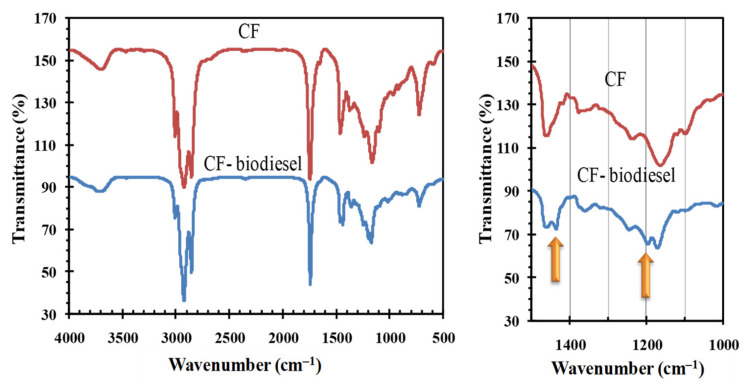
FTIR spectra of CF and CF-biodiesel.

The FTIR spectra of CF and CF-derived biodiesel are shown in Figure 12. The spectra lying within 1850–950 cm^−1^ are almost alike since the chemical groups in the CF and CF-biodiesel are essentially similar. However, there are some discernible disparities in spectra within 1500–1000 cm^−1^. In particular, the C–O stretching vibrations in CF engender a peak at 1163 cm^−1^, while the C–O stretching vibrations in biodiesel engender two peaks at 1169 cm^−1^ and 1195 cm^−1^. The presence of CH_3_ groups next to the carbonyl groups in FAME explains the peak at 1195 cm^−1^ [63,64,65]. Furthermore, the spectra exhibit two peaks, one at 1375 cm^−1^ in CF and another at 1436 cm^−1^ in biodiesel corresponding to CH_2_ and CH_3_ bending vibrations, respectively, demonstrating that CF has finally converted into biodiesel.

**Figure 13:**
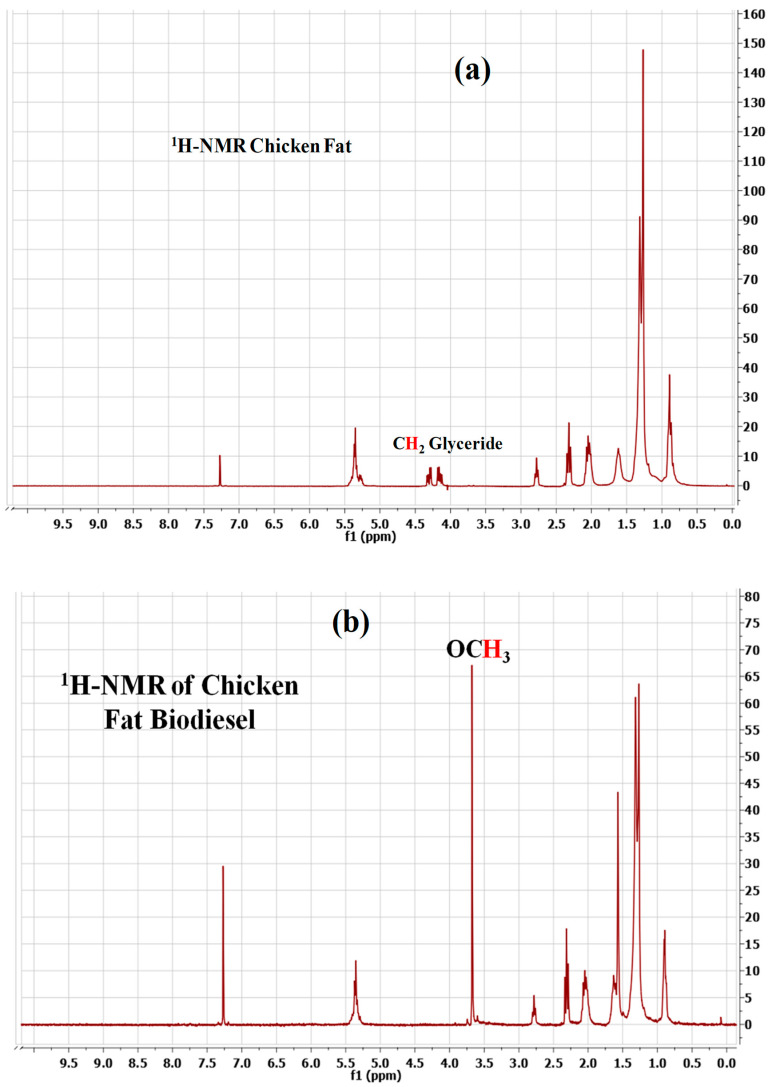
^1^H NMR spectra of: (**a**) CF, and (**b**) CF-derived biodiesel.

The ^1^H NMR spectra of CF and CF-derived fatty acid methyl esters (FAME) have been shown in Figure 13a,b, respectively. The characteristic peaks of the proton of the methyl ester showed up as a singlet at 3.667 ppm from methoxy protons and a triplet of the α-carbonyl methylene proton at 2.306 ppm. These two offbeat peaks corroborate the realization of the methyl ester in the biodiesel via transesterification. The signature of unsaturated FAMEs in the biodiesel is evident from the proton peak of olefins at 5.35 ppm. Similarly, the signature of saturated FAMEs in the biodiesel can be detected in the spectra via (i) the highly intense peaks of methylene protons at 1.308 ppm signifying the realization of the long chain of methylene groups, and (ii) the peak of terminal methyl protons at 0.871 ppm. The multiplet at 1.6 ppm is associated with the β-carbonyl methylene proton. Moreover, the spectra do not exhibit any peak at around 4 ppm corresponding to proton glycerides which signifies that glycerol is absent in our produced biodiesel [58] and thus, it is of high quality.

## 4. Conclusions

1. In this work, we synthesized a heterogeneous carbon-based sulfonated tire polymer char TPC-SO_3_H solid acid catalyst from scrap tire through a two-step procedure comprising pyrolysis of scrap tire at a temperature of 500 °C followed by thermal acid treatment employing concentrated H_2_SO_4_ at 180 °C.
2. The produced catalyst was then successfully utilized to synthesize biodiesel from waste chicken fat through a two-step conversion process of esterification to decrease the FFA content of chicken fat, followed by conventional alkali-catalyzed transesterification.
3. The optimal esterification reaction conditions found were 70 °C and 120 min with a 15:1 methanol-to-CF molar ratio and 5 wt.% catalyst dosage.
4. The synthesized catalyst performed well for the first three cycles without exhibiting any significant degradation in its catalytic activity under the ascertained optimal settings.
5. The chemical and physicochemical properties of the synthesized biodiesel were found to fall under the set limits of biodiesel, suggesting the complete conversion of chicken fat into biodiesel.
6. Lastly, we compared our scrap-tire derived catalyst with other previously reported waste-derived sulfonated catalysts and concluded that our synthesized catalyst produced the maximal biodiesel yield.

## Figures and Tables

**Table 1:** Activity comparison of various waste-derived carbon-based sulfonated catalysts for biodiesel synthesis.

Waste Raw Material	Sulfonating Agent	Reaction Conditions				Conversion Efficiency (%)	Ref.
		Temp. (°C)	Time (Minutes)	Molar Ratio	Catalyst (wt.%)		
Coffee Residue	Concentrated H_2_SO_4_	60	240	3:1	5	71.5	[56]
Sugar Cane Bagasse	Concentrated H_2_SO_4_	170	30	20:1	11.5	80	[34]
Coconut Meal Residue	Concentrated H_2_SO_4_	65–70	720	12:1	5	92.7	[44]
Corn Straw	Concentrated H_2_SO_4_	60	240	7:1	7	93	[57]
Cacao Shell	Concentrated H_2_SO_4_	42	1440	7:1	5	93	[58]
Sugar Cane Bagasse	Concentrated H_2_SO_4_	66	240	18:1	1	94.4	[59]
D-glucose	Concentrated H_2_SO_4_	65	134	12.2:1	2.9	94.5	[43]
Murumuru Kernel Shells	Concentrated H_2_SO_4_	90	90	10:1	5	97.2	[60]
Bamboo	Concentrated H_2_SO_4_	65	120	10:1	10	97.3	[61]
Tire Polymer Waste	Concentrated H_2_SO_4_	70	120	15:1	5	98.8	Present Study

**Table 2:** The physicochemical properties of the biodiesel obtained from CF-biodiesel characterization.

S. No.	Parameters	Unit	FAME	EN 14214
1	Flash Point	°C	152	100–170
2	Cloud Point	°C	0	-
3	Pour Point	°C	−2	−5–10
4	Kinematic Viscosity (40 °C)	cSt	4.68	1.9–6.0
5	Density (31 °C)	Kg mm^−3^	881	860–900
6	Acid Value	mg KOH g^−1^	0.5	0.8
7	Ash Content	wt.%	Nil	0.02
8	Iodine Index	g I_2_ 100 g^−1^	120	-

## Data Availability

The data presented in this study are available from the corresponding author upon reasonable request.
